# Ozone Inhalation Provokes Glucocorticoid-Dependent and -Independent Effects on Inflammatory and Metabolic Pathways

**DOI:** 10.1093/toxsci/kfw061

**Published:** 2016-04-01

**Authors:** Errol M. Thomson, Shinjini Pal, Josée Guénette, Michael G. Wade, Ella Atlas, Alison C. Holloway, Andrew Williams, Renaud Vincent

**Affiliations:** * *Environmental Health Science and Research Bureau, Health Canada, Ottawa, Ontario, K1A 0K9, Canada and; * *Environmental Health Science and Research Bureau, Health Canada, Ottawa, Ontario, K1A 0K9, Canada and; * *Environmental Health Science and Research Bureau, Health Canada, Ottawa, Ontario, K1A 0K9, Canada and; * *Environmental Health Science and Research Bureau, Health Canada, Ottawa, Ontario, K1A 0K9, Canada and; * *Environmental Health Science and Research Bureau, Health Canada, Ottawa, Ontario, K1A 0K9, Canada and; ^†^ Department of Obstetrics and Gynecology, McMaster University, Hamilton, Ontario, L8N 3Z5, Canada; * *Environmental Health Science and Research Bureau, Health Canada, Ottawa, Ontario, K1A 0K9, Canada and; * *Environmental Health Science and Research Bureau, Health Canada, Ottawa, Ontario, K1A 0K9, Canada and

**Keywords:** air pollution, ozone, glucocorticoid, stress response, hypothalamic–pituitary–adrenal (HPA) axis, systemic effects, inflammation, metabolic effects.

## Abstract

Growing evidence implicates air pollutants in adverse health effects beyond respiratory and cardiovascular disease, including metabolic impacts (diabetes, metabolic syndrome, obesity) and neurological/neurobehavioral outcomes (neurodegenerative disease, cognitive decline, perceived stress, depression, suicide). We have shown that inhalation of particulate matter or ozone activates the hypothalamic–pituitary–adrenal axis in rats and increases plasma levels of the glucocorticoid corticosterone. To investigate the role of corticosterone in mediating inflammatory and metabolic effects of pollutant exposure, in this study male Fischer-344 rats were administered the 11β-hydroxylase inhibitor metyrapone (0, 50, 150 mg/kg body weight) and exposed by nose-only inhalation for 4 h to air or 0.8 ppm ozone. Ozone inhalation provoked a 2-fold increase in plasma corticosterone, an effect blocked by metyrapone, but did not alter epinephrine levels. Inhibition of corticosterone production was associated with increased inflammatory signaling in the lungs and plasma in response to ozone, consistent with a role for glucocorticoids in limiting local and systemic inflammatory responses. Effects of ozone on insulin and glucagon, but not ghrelin or plasminogen activator inhibitor-1, were modified by metyrapone, revealing glucocorticoid-dependent and -independent effects on circulating metabolic and hemostatic factors. Several immunosuppressive and metabolic impacts of ozone in the lungs, heart, liver, kidney, and spleen were blocked by metyrapone and reproduced through exogenous administration of corticosterone (10 mg/kg body weight), demonstrating glucocorticoid-dependent effects in target tissues. Our results support involvement of endogenous glucocorticoids in ozone-induced inflammatory and metabolic effects, providing insight into potential biological mechanisms underlying health impacts and susceptibility.

A growing number of studies support the contention that health effects of air pollutants extend beyond the pulmonary and cardiovascular systems. These include metabolic disorders such as metabolic syndrome (Park *et al.*, 2010), obesity (Rundle *et al.*, 2012), and type 2 diabetes (Andersen *et al.*, 2012; Brook *et al.*, 2008; Brook *et al.*, 2013; Kramer *et al.*, 2010; Raaschou-Nielsen *et al.*, 2013), and neurological and neurobehavioral disorders such as cognitive decline (Chen and Schwartz 2009; Power *et al.*, 2011; Ranft *et al.*, 2009; Weuve *et al.*, 2012), hyperactivity (Newman *et al.*, 2013), perceived stress (Mehta *et al.*, 2015), depression (Lim *et al.*, 2012; Szyszkowicz *et al.*, 2009), and suicide (Kim *et al.*, 2010; Szyszkowicz *et al.*, 2010). The societal burden of metabolic disorders and mental illness is enormous and growing (Murray *et al.*, 2012). For example, 20% of the Canadian population exhibits characteristics of metabolic syndrome (Riediger and Clara, 2011), a significant risk factor for diabetes and cardiovascular disease. Given the prevalence of these disease states, and the near ubiquitous exposure of the population to air pollutants, even modest air pollutant-induced increases in disease incidence may have significant implications for public health.

Metabolic impacts of pollutants are emerging issues in air pollution research, and underlying mechanisms remain poorly understood (Hutcheson and Rocic, 2012; Liu *et al.*, 2013). That adverse health effects are associated with multiple pollutants may relate to a more general response to pollutant inhalation, differential sensitivity to specific pollutants in the populations examined, or to the fact that air pollution is a complex mixture of constituent toxicants. Results from animal studies in general support the notion of pollutant impacts on metabolic function. For example, 24 week exposure to concentrated ambient particles increased insulin resistance, visceral fat, and systemic and cellular inflammatory markers in rats fed a high-fat diet (Sun *et al.*, 2009). Cardiovascular impacts of repeated exposure to ozone and fine particulate matter were exacerbated in a high-fructose diet-induced metabolic syndrome rat model (Wagner *et al.*, 2014). Short-term exposure studies have also investigated potential mechanisms underlying metabolic effects. Acute exposure to ozone has been shown to reduce glucose tolerance in rats (Bass *et al.*, 2013), alter serum metabolic profiles (Miller *et al.*, 2015), and activate stress centers in the brain and pituitary (Gackiere *et al.*, 2011; Thomson *et al.*, 2007, 2013).

We have shown that short-term inhalation of ozone or particulate matter rapidly increases plasma levels of the hypothalamic–pituitary–adrenal (HPA) axis stress hormones adrenocorticotropic hormone and the glucocorticoid corticosterone in rats (Thomson *et al.*, 2013). Glucocorticoids (corticosterone in rodents, cortisol in humans) regulate a large number of processes that include immune response, glucose metabolism, adipocyte differentiation, and the action of other hormones. Transient HPA axis activation in response to a stressor is a critical adaptive response in healthy individuals. However, repeated or chronic stress can lead to dysregulation of the HPA axis, associated with metabolic, neurobehavioral, cardiovascular, and reproductive disease processes (Anagnostis *et al.*, 2009; Chrousos and Kino, 2009). Chronic administration of corticosterone in conjunction with a high-fat diet produces a model of metabolic syndrome or type 2 diabetes (Shpilberg *et al.*, 2012), establishing a causal relationship between glucocorticoids and metabolic dysfunction. Given the overlap between conditions resulting from HPA axis dysfunction and those associated with air pollution, we hypothesize that chronic activation and dysfunction of the stress response system could contribute to adverse health effects of pollutants (Thomson, 2014).

This study is based on the hypothesis that glucocorticoids mediate downstream effects of pollutant inhalation on inflammatory and metabolic systems. To distinguish between glucocorticoid-dependent and -independent processes, we studied the impact of pharmacological blockade of corticosterone synthesis on responses to ozone inhalation in the lungs, on metabolic and inflammatory biomarkers in circulation, and on effects in extrapulmonary tissues. While recognizing that environmental exposures are invariably to mixtures of pollutants, ozone provides a useful model for assessment of secondary (indirect) effects of pollutant exposure, as this highly reactive gas is entirely consumed in the lungs. Adverse health impacts of ozone are a significant population health concern, with standards for ozone regularly exceeded and levels expected to increase as a result of global warming and greater release of precursor chemicals. Our results demonstrate that ozone inhalation produces glucocorticoid-dependent and -independent effects on inflammatory and metabolic pathways, providing insight into potential biological mechanisms underlying the adverse health effects associated with air pollutants.

## MATERIALS AND METHODS

### Animals

Specific pathogen-free male Fischer-344 rats (200–250 g) were obtained from Charles River (St. Constant, Québec, Canada). Animals were housed in individual plexiglass cages on wood-chip bedding under HEPA-filtered air and held to a 12 h dark/light cycle (lights on 06:00 to 18:00). Food and water were provided *ad libitum*. All experimental protocols were reviewed and approved by the Animal Care Committee of Health Canada.

### Inhalation exposure to air pollutants

Animals were trained for progressively longer periods (0–4 h) in nose-only exposure tubes over 7 consecutive days for acclimatization. Rats (n = 5/group) were administered metyrapone (50, 150 mg/kg; Sigma-Aldrich Canada Co., Oakville, Ontario, Canada) or vehicle (40% propylene glycol in buffered saline) by subcutaneous injection 1 h prior to exposure, a dose-range and time-point shown to block the stress-induced rise in corticosterone (Li *et al.*, 2009; Marinelli *et al.*, 1996; Sillence and Rodway, 1987), and then exposed for 4 h to clean air or to 0.8 ppm ozone. The rationale for the level of ozone exposure used and dosimetric comparison of rats and humans has been presented elsewhere (Thomson *et al.*, 2005). A separate group of animals (n = 5) was administered corticosterone (10 mg/kg in the same vehicle; Sigma-Aldrich) by subcutaneous injection 1 h prior to exposure, and exposed to clean air for 4 h. Exposures were conducted in the morning commencing at 08:00, with necropsies performed in the early afternoon. Experiments were conducted across 5 days, with 1 animal per treatment group exposed on each day. The timing of exposure and necropsy was balanced across all groups to avoid any potential bias due to diurnal variation in corticosterone levels. Parallel exposures to air and to ozone were conducted using single-tier nose-only exposure manifolds (CH Technologies, Ann Arbor, Michigan). Both manifolds (air and ozone treatments) used the same source (12 lpm HEPA-filtered air) for providing air to the animals. In the ozone system, a silent arc generator (Erwin Sander, Uetze, Germany) made ozone from medical-grade oxygen. A feedback loop (Guenette *et al.*, 1997) maintained a steady ozone concentration of 0.8 ppm (average 0.799 ± 0.033 ppm) by measuring ozone concentration (TECO model 49, C. D. Nova-Tech Inc., Markham, Ontario) at 1 exposure port and adjusting the ozone bypass being mixed with the main airstream. Slightly negative pressure (approximately 125 Pa) was maintained by continuously removing air from the nose-only manifold’s exhaust. The system achieved steady state before exposures started. Animals were euthanized immediately after the 4 h exposure, along with naïve animals (n = 5) that remained in cages rather than being introduced into the nose-only exposure system.

### Biological samples

Rats were anaesthetized by administration of isoflurane (5% at 1.5 L of O_2_/min). Blood was collected from the abdominal aorta into vacutainer tubes containing EDTA at 10 mg/ml and phenyl methyl sulfonyl fluoride at 1.7 mg/ml. Plasma was isolated by centrifugation (1448 × g for 10 min), aliquoted, and frozen at −80°C. The lungs were washed by bronchoalveolar lavage with warm saline (37°C) at 30 ml/kg body weight. Lavage fluid was centrifuged (400 × g for 10 min at 4 °C) to remove cells and frozen at −80 °C. Lung, heart, liver, kidney, and spleen samples were snap-frozen in liquid nitrogen.

### Ex vivo corticosterone production assay

To evaluate adrenal production of corticosterone in response to treatments, 1 adrenal gland from each animal was rapidly dissected and incubated whole as previously described (Carsia *et al.*, 1997). Briefly, glands were rapidly trimmed of connective tissue and incubated for 2 h at 37°C in 2 ml of prewarmed serum-free Eagle’s Minimum Essential Medium supplemented with sodium pyruvate and L-glutamine (2 mM each; Thermo-Fisher, Nepean, Ontario) and with 0.1% fatty-acid free bovine serum albumin (Sigma-Aldrich). Media was collected after 2 h and stored at −80°C until assayed for corticosterone production as described below.

### Circulating stress, inflammatory, and metabolic factors

Corticosterone was assessed using the DetectX Corticosterone Enzyme Immunoassay Kit (Arbor Assays, Ann Arbor, Michigan). Plasma epinephrine was measured using the Rat Epinephrine ELISA Kit CUSABIO, Wuhan, China). Insulin was assessed using the Rat/Mouse Insulin ELISA kit (Millipore Canada Ltd., Etobicoke, Ontario). Cholesterol, HDL, and LDL were assessed using the ABX Pentra 400 (Horiba Instruments Inc., Irvine, California). Cytokines were assessed using the Bio-Plex Pro Rat Cytokine 24-plex Assay kit and metabolic markers (ghrelin, glucagon-like peptide-1, glucagon, leptin, plasminogen activator inhibitor [PAI]-1) were measured with the Bio-Plex Pro Rat Diabetes 5-plex Assay kit (Bio-Rad Laboratories Ltd., Mississauga, Ontario, Canada). All kits were run according to manufacturer protocols.

### Gene expression

Frozen lung, heart, liver, kidney, and spleen samples were homogenized in TRIzol reagent (Invitrogen Canada Inc., Burlington, Ontario, Canada), and total RNA was isolated according to manufacturer instructions. RNA was quantified using the RiboGreen RNA Quantitation Reagent and Kit (Molecular Probes, Eugene, Oregon). Total RNA was reverse transcribed using MuLV reverse transcriptase and random hexamers (Applied Biosystems, Mississauga, Ontario, Canada) according to manufacturer instructions. In parallel, RNA was incubated with all components of the reagent mix with the exception of reverse transcriptase to generate negative control samples for assessment of contaminating genomic DNA in subsequent PCR analyses. Primers (Supplementary Data) were designed to produce amplicons with an optimal annealing temperature of 60°C using the Universal Probe Library design software (Roche Diagnostics Canada, Laval, Québec, Canada), and Primer-BLAST software (National Center for Biotechnology Information, Bethesda, Maryland), and high-efficiency reactions (>90% efficiency) were verified across a dilution range of rat cDNA. Twenty nanograms of cDNA were incubated with iQ SYBR Green Supermix (Bio-Rad Laboratories Ltd.) and 200 nM of each primer in a total volume of 10 μl/well. All reactions were performed in duplicate on 384-well plates in a spectrofluorometric thermal cycler (Lightcycler 480, Roche Diagnostics Canada). PCR runs were initiated by incubation at 95°C for 3 min to activate the iTAQ polymerase followed by 50 cycles of denaturation at 95°C, annealing at 60°C, and elongation at 72°C, each for 10 s. Fluorescence was monitored at every cycle during the elongation step. A melt curve analysis was conducted following each run to verify product purity. Expression was calculated relative to appropriate reference genes using the delta-delta Ct method (Livak and Schmittgen, 2001). A panel of 4 reference genes (β-actin, GAPDH, PP1A, YWHAZ) was assessed for stability across treatment groups using RefFinder (Chen *et al.*, 2011) and ANOVAs as previously described (Thomson *et al.*, 2013). On the basis of this analysis, PP1A was selected for normalization of gene expression in the lungs, GAPDH and PP1A for the heart, liver, and spleen, and GAPDH and β-actin for the kidney.

### Statistical analyses

A block factorial design was employed to assess the effects of ozone and metyrapone. Data (excluding naive and exogenous corticosterone groups) were analyzed by two-way ANOVA with OZONE (0, 0.8 ppm) and MET (0, 50, 150 mg/kg metyrapone) as factors, followed by the Holm-Sidak multiple comparison procedure to elucidate the pattern of significant effects (α = .05; Sigma-Plot 12.3, Systat Software Inc., San Jose, California and R statistical package [R Core Team, 2015]). Estimates for the log fold change and standard error were calculated for all pairwise comparisons, with the estimate for the standard error obtained using the delta method (Casella and Berger, 2001). Where necessary, results were transformed to meet the requirements of normality and equal variance. Differences between the exogenous corticosterone and vehicle air control groups were assessed by *t* test (Sigma-Plot 12.3).

## RESULTS

### Effects of Ozone and Metyrapone on Plasma Corticosterone

**FIG. 1.**
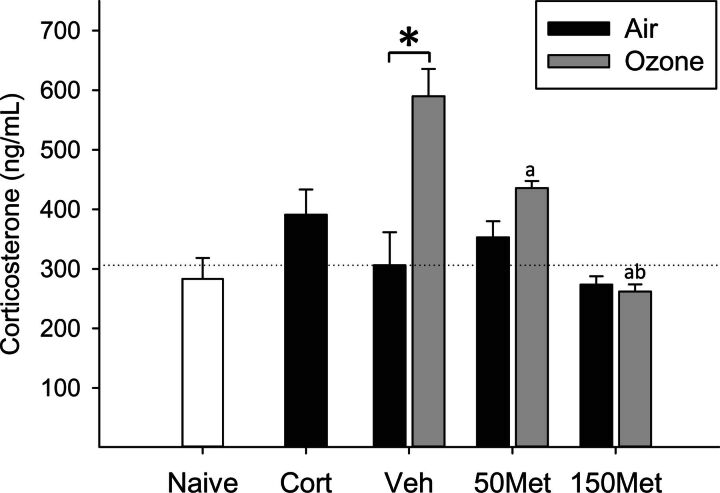
.Effects of ozone and metyrapone on plasma corticosterone. Animals were treated with vehicle (Veh) or metyrapone (Met; 50, 150 mg/kg) and exposed for 4 h to clean air or 0.8 ppm ozone as described in the Materials and Methods. Corticosterone (Cort; 10 mg/kg) was administered to a separate set of animals and exposed to air, and naïve animals remained in their cages. Each bar represents mean ± SE (n = 5/group). OZONE × MET interaction, *P* < .001 (two-way ANOVA). Symbols above bars denote statistical significance of pairwise comparisons (*P* < .05, Holm-Sidak) as follows: (*) Air versus Ozone within vehicle; (a) 50 or 150 versus 0 mg/kg metyrapone within Ozone; (b) 150 versus 50 mg/kg metyrapone within Ozone

To investigate involvement of glucocorticoids in mediating downstream effects of pollutant exposure, Fischer-344 rats were administered vehicle or the drug metyrapone, which has been previously shown to block stress-induced increases in glucocorticoid synthesis (Li *et al.*, 2009; Marinelli *et al.*, 1996; Sillence and Rodway, 1987), and exposed to clean air or ozone. Ozone inhalation produced a significant 2-fold increase in plasma corticosterone immediately after exposure that was prevented by metyrapone (OZONE × MET interaction, *P* < .001; Figure 1). Animals administered exogenous corticosterone tended to have slightly higher corticosterone levels than the air control group (+20%, Vehicle Air vs CORT group, *P* = .12; Figure 1), and ex vivo production of corticosterone from the adrenal gland was significantly reduced (−60%, Vehicle Air vs CORT group, *P* = .04; Supplementary Data), consistent with negative feedback regulation and confirming delivery of a biologically effective dose of corticosterone. Plasma epinephrine was not significantly altered by ozone nor modified by metyrapone (data not shown).

### Inflammatory and Antioxidant Response in the Lungs

**FIG. 2.**
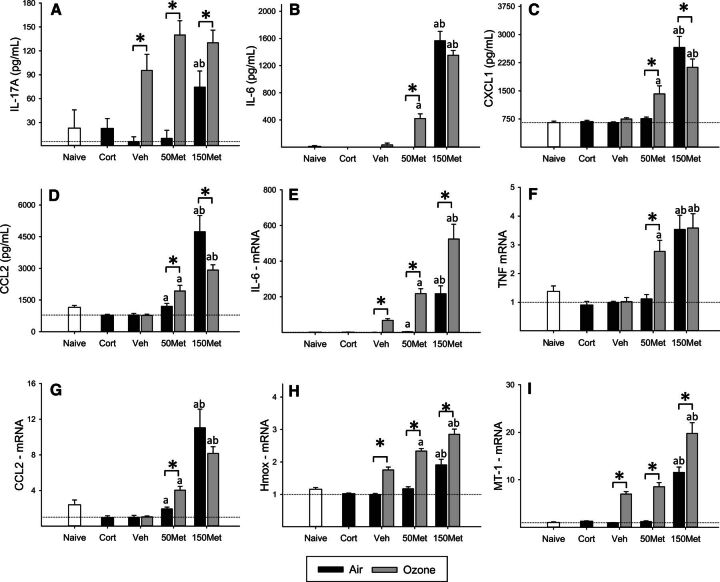
Inflammatory and antioxidant response in the lungs. Animals were administered metyrapone (Met; 50, 150 mg/kg) or vehicle (Veh), exposed to clean air or 0.8 ppm ozone for 4 h, and euthanized immediately after exposure. Corticosterone (Cort; 10 mg/kg) was administered to a separate set of animals and exposed to air, and naïve animals remained in their cages. Cytokines were recovered by bronchoalveolar lavage (BAL) and assessed by multiplex immunoassay, and gene expression was measured in lung tissue by real-time PCR. Data were assessed for significant factor interactions or main effects by two-way ANOVA, followed by Holm-Sidak pairwise comparison. Complete statistical analyses are presented in Supplementary Data. Each bar represents mean ± SE (n = 5/group). A, IL-17A. OZONE main effect, *P* = .013; MET main effect, *P* < .001. B, IL-6. OZONE × MET interaction, *P* < .001. C, CXCL1. OZONE × MET interaction, *P* = .01. D, CCL2. OZONE × MET interaction, *P* = .003. E, IL-6 mRNA. OZONE × MET interaction, *P* < .001. F, TNF mRNA. OZONE × MET interaction, *P* < .001. G, CCL2 mRNA. OZONE × MET interaction, *P* = .007. H, HMOX mRNA. OZONE × MET interaction, *P* = .027. I, MT-1 mRNA. OZONE × MET interaction, *P* < .001. J, MT-2 mRNA. OZONE main effect, *P* < .001; MET main effect, *P* < .001. Symbols above bars denote statistical significance of pairwise comparisons (*P* < .05, Holm-Sidak) as follows: (*) Air versus Ozone within each drug dose; (a) 50 or 150 versus 0 mg/kg metyrapone within Air or Ozone; (b) 150 versus 50 mg/kg metyrapone within Air or Ozone.

Short-term exposure to 0.8 ppm ozone causes epithelial injury in terminal bronchioles and alveolar ducts, and tends to initially decrease cell recovery by bronchoalveolar lavage, with subsequent influx of inflammatory cells that typically peaks 24–48 h after injury (eg Vincent and Adamson, 1995; Zhao *et al.*, 1998). Consistent with these previous results, cell numbers recovered in bronchoalveolar lavage fluid immediately after the 4 h exposure were slightly reduced in ozone-exposed animals, and this effect was not altered by metyrapone (OZONE main effect, *P* < .001, data not shown). To investigate early effects of ozone inhalation on inflammatory signaling and antioxidant response in the lungs and their modification by metyrapone treatment, we examined inflammatory mediators in bronchoalveolar lavage fluid and lung gene expression. Exposure to ozone increased levels of the neutrophil chemoattractant interleukin (IL)-17A in bronchoalveolar lavage fluid (OZONE main effect, *P* = .013; Figure 2A) independent of drug treatment (MET main effect, *P* < .001). In contrast, IL-6, chemokine (C-X-C motif) ligand 1 (CXCL1; also known as GRO/KC), and chemokine (C-C) motif ligand 2 (CCL2; also known as monocyte chemotactic protein-1) were increased by ozone only in the lungs of animals treated with 50 mg/kg body weight metyrapone (all OZONE × MET interactions, *P* < .01; Figs 2B–D). Baseline levels of inflammatory mediators in air-exposed animals were substantially increased at the 150 mg/kg body weight dose of metyrapone, with variable effects of ozone. The mRNA levels of inflammatory mediators exhibited a similar pattern of effects (Figs 2E–G), with metyrapone treatment increasing the response to ozone for IL-6 (OZONE × MET, *P* < .001), tumor necrosis factor (TNF) (OZONE × MET, *P* < .001), and CCL2 (OZONE × MET, *P* = .008). Ozone increased lung mRNA levels of the antioxidant factors heme oxygenase (HMOX)-1 (OZONE main effect, *P* < .001; Figure 2H) and metallothionein (MT)-1 (OZONE main effect, *P* < .001; Figure 2I), effects that were not significantly modified by metyrapone treatment despite an overall increase in baseline transcript levels at the highest dose (all MET main effect, *P* < .001).

### Effects on Plasma Cytokines and Chemokines

**FIG. 3.**
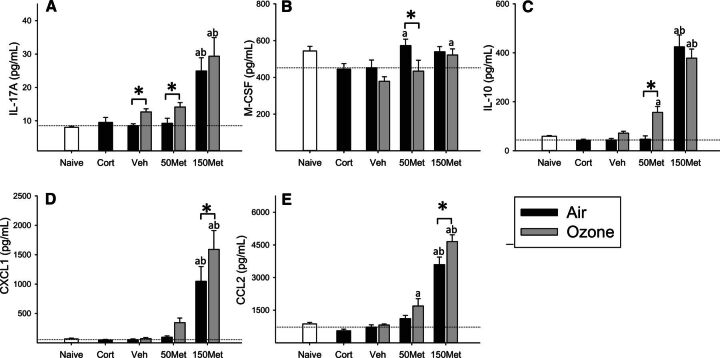
Effects of ozone and metyrapone on plasma cytokines. Cytokine levels were assessed in the plasma of animals administered vehicle (Veh) or metyrapone (Met; 50, 150 mg/kg) and exposed to air or 0.8 ppm ozone for 4 h. Corticosterone (Cort; 10 mg/kg) was administered to a separate set of animals and exposed to air, and naïve animals remained in their cages. Data were assessed for significant factor interactions or main effects by two-way ANOVA, followed by Holm-Sidak pairwise comparison. Complete statistical analyses are presented in Supplementary Data. Each bar represents mean ± SE (n = 5/group). A, IL-17A. OZONE main effect, *P* = .006; MET main effect, *P* < .001. B, M-CSF. OZONE main effect, *P* = .024; MET main effect, *P* = .018. C, IL-10. OZONE × MET interaction, *P* = .032; D, CXCL1. OZONE main effect, *P* = .002; MET main effect, *P* < .001. E, CCL2. OZONE main effect, *P* = .008; MET main effect, *P* < .001. Symbols above bars denote statistical significance of pairwise comparisons (*P* < .05, Holm-Sidak) as follows: (*) Air versus Ozone within each drug dose; (a) 50 or 150 versus 0 mg/kg metyrapone within Air or Ozone; (b) 150 versus 50 mg/kg metyrapone within Air or Ozone.

Assessment of 24 cytokines in plasma revealed little effect of exposure to ozone alone beyond a small increase in IL-17A (OZONE main effect, *P* = .006; Figure 3A) and decrease of macrophage colony stimulating factor (M-CSF; OZONE main effect, *P* = .024; Figure 3B) compared to corresponding drug-matched air groups, with metyrapone independently increasing levels of both factors (MET main effect, *P* < .05). Administration of metyrapone tended to increase effects of ozone on IL-10 (OZONE × MET interaction, *P* = .03; Figure 3C), CXCL1 (OZONE main effect, *P* = .002; MET main effect, *P* < .001; Figure 3D) and CCL2 (OZONE main effect, *P* = .008; MET main effect, *P* < .001; Figure 3E).

### Effects on Plasma Metabolic Biomarkers

Effects of ozone and metyrapone on metabolic hormones were mixed (Table 1). Ozone and metyrapone both significantly decreased plasma glucagon, with the effect of ozone prevented at the high dose of metyrapone (OZONE × MET interaction, *P* = .045). Insulin levels also tended to be lower after ozone exposure (not statistically significant), but were unchanged by ozone at 50 mg/kg metyrapone; insulin levels were significantly decreased at 150 mg/kg metyrapone, with ozone increasing insulin to near control levels (OZONE × MET interaction, *P* = .03). Ghrelin was decreased by ozone (OZONE main effect, *P* < .001) and increased by metyrapone (MET main effect, *P* < .001), with neither factor significantly affecting leptin. Levels of the antifibrinolytic factor PAI-1 were independently increased by ozone and metyrapone (OZONE main effect, *P* = .046; MET main effect, *P* < .001). Ozone inhalation did not significantly alter cholesterol, LDL, and HDL levels (data not shown).

**TABLE 1.** Plasma Metabolic Markers

Drug	Exposure	Insulin	Glucagon	GLP-1	Ghrelin	Leptin	PAI-1
		pg/ml	pg/ml	pg/ml	pg/ml	pg/ml	pg/ml
Naïve	n/a	1330 ± 224	1061 ± 72	237 ± 30	13 229 ± 1431	3011 ± 220	229 ± 23
Cort	Air	1063 ± 428	1121 ± 185	218 ± 28	18 059 ± 1048	3308 ± 684	319 ± 30
Veh	Air	1169 ± 408	895 ± 104	164 ± 47	19 421 ± 1570	3450 ± 497	302 ± 29
	Ozone	657 ± 46	455 ± 76*	203 ± 20	13 525 ± 989*	3994 ± 443	350 ± 21
50Met	Air	931 ± 112	650 ± 123	234 ± 35	25 449 ± 1322	3617 ± 393	294 ± 30
	Ozone	994 ± 118	362 ± 61	238 ± 37	20 757 ± 2862	4269 ± 616	389 ± 19*
150Met	Air	445 ± 170	498 ± 114	246 ± 31	56 314 ± 12 079	4319 ± 493	593 ± 45
	Ozone	1114 ± 307*	592 ± 124	339 ± 58	31 265 ± 1524*	4495 ± 771	611 ± 35

Levels of each endpoint were measured in the plasma of animals administered vehicle (Veh) or metyrapone (Met; 50, 150 mg/kg) and exposed to air or 0.8 ppm ozone for 4 h. Corticosterone (Cort; 10 mg/kg) was administered to a separate set of animals and exposed to air, and naïve animals remained in their cages. Values indicate group means ± SE (n = 5/group). Data were assessed for significant factor interactions or main effects by two-way ANOVA, followed by Holm-Sidak pairwise comparison. For simplicity, only significant Air versus Ozone pairwise comparisons within each drug treatment group are indicated. Complete statistical analyses, including evaluation of metyrapone effects, are presented in Supplementary Data.

*P < .05, Holm-Sidak pairwise comparison (air vs ozone).

### Tissue-Level Effects of Ozone Exposure and Metyrapone Treatment

**FIG. 4.**
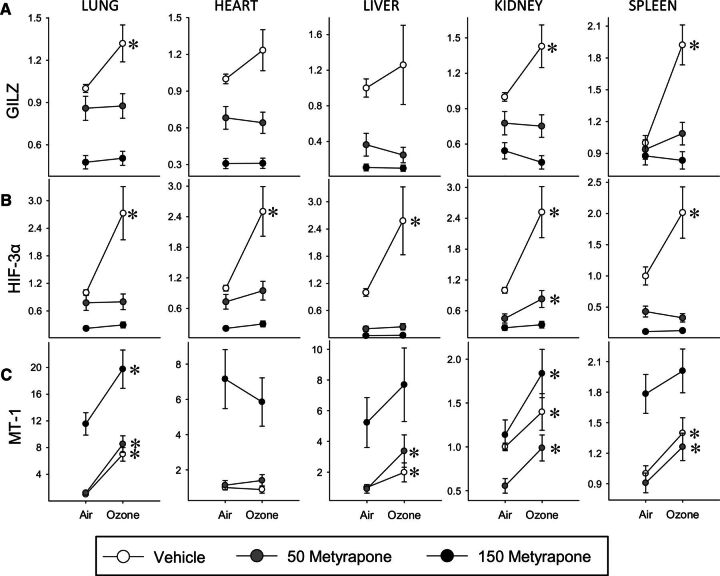
Tissue-level effects of ozone and metyrapone on the expression of inflammatory, metabolic, and antioxidant genes. The mRNA levels were assessed in tissues of animals exposed to air or 0.8 ppm ozone following administration of vehicle or metyrapone (Met; 50, 150 mg/kg). Values are expressed as mRNA fold-change relative to vehicle air control group ± SE (n = 5/group). Data were assessed for significant factor interactions or main effects by two-way ANOVA, followed by Holm-Sidak pairwise comparison. For simplicity, only significant Air versus Ozone pairwise comparisons are indicated (*P < .05). Complete statistical analyses are presented in Supplementary Data. A, Glucocorticoid-inducible leuzine zipper (GILZ). Lung: MET main effect, *P* < .001, OZONE main effect, *P* = .051. Heart: MET main effect, *P* < .001. Liver: MET main effect, *P* < .001. Kidney: MET × OZONE interaction, *P* = .020. Spleen: MET × OZONE interaction, *P* = .009. B, Hypoxia-inducible factor (HIF)-3α. Lung: MET × OZONE interaction, *P* < .001. Heart: MET main effect, *P* < .001, OZONE main effect, *P* < .001. Liver: MET main effect, *P* < .001, OZONE main effect, *P* = .016. Kidney: MET main effect, *P* < .001, OZONE main effect, *P* < .001. Spleen: MET × OZONE interaction, *P* = .01. C, Metallothionein (MT)-1. Lung: MET main effect, *P* < .001, OZONE main effect, *P* < .001. Heart: MET main effect, *P* < .001. Liver: MET main effect, *P* < .001, OZONE main effect, *P* < .001. Kidney: MET main effect, *P* < .001, OZONE main effect, *P* < .001. Spleen: MET main effect, *P* < .001, OZONE main effect, *P* < .001.

To evaluate the role of corticosterone in mediating tissue-level effects of ozone, we examined expression of genes implicated in inflammatory, metabolic, and antioxidant responses in the lungs, heart, liver, kidney, and spleen. Glucocorticoid-inducible leuzine zipper (GILZ), an important regulator of the anti-inflammatory impacts of glucocorticoids (Ayroldi and Riccardi, 2009), was increased across organs by ozone inhalation, and this effect was blocked by metyrapone (Figure 4A). In accordance with these observations, expression of inflammatory genes (IL-1β, TNF, CCL2) tended to decrease in response to ozone in most organs except the kidney, effects that were predominantly blocked or reduced by metyrapone (Supplementary Data). Levels of hypoxia inducible factor (HIF)-3α, involved in the response to hypoxia and glucoprivation (Heidbreder *et al.*, 2007) were uniformly increased across organs by ozone and blocked by metyrapone treatment (Figure 4B). In contrast, mRNA levels of two other genes involved in metabolic processes, BCL2/adenovirus E1B 19 kDa-interacting protein (BNIP)-3, a stress-responsive protein involved in mitochondrial function and lipid metabolism (Glick *et al.*, 2012), and sterol regulatory element-binding protein (SREBP)-1, a transcription factor involved in cholesterol and fatty acid biosynthesis and uptake and insulin signaling (Horton *et al.*, 2002), were altered by ozone and metyrapone in a tissue-dependent manner (Supplementary Data). Ozone inhalation increased mRNA levels of the antioxidant and glucocorticoid-responsive factor MT-1, an effect that was not blocked by metyrapone (Figure 4C). The pattern of response of a second metallothionein gene (MT-2) was entirely consistent with MT-1 (Supplementary Data). In addition to regulation by ozone, GILZ, HIF-3α, and MT mRNA levels were impacted by metyrapone in a dose-dependent manner, as indicated by significant MET main effects or OZONE × MET factor interactions (Supplementary Data).

### Reproduction of Ozone Effects Through Administration of Exogenous Corticosterone

**FIG. 5.**
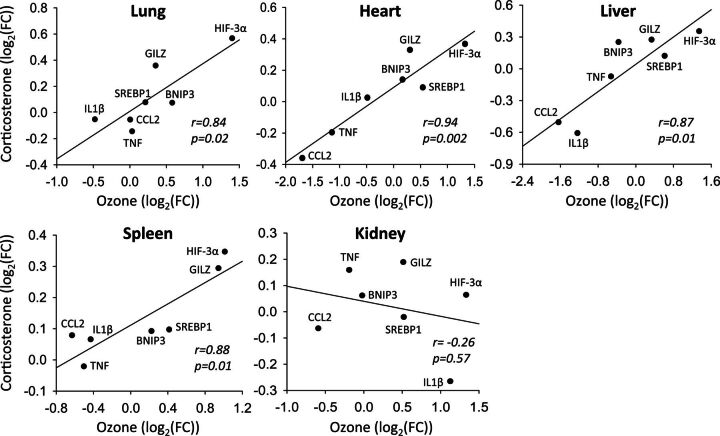
Comparison of transcriptional responses to ozone and exogenous corticosterone. The mRNA levels of immune response (glucocorticoid-inducible leucine zipper (GILZ), chemokine (C-C motif) ligand 2 (CCL2), interleukin (IL)-1β, tumor necrosis factor (TNF)) and metabolic (hypoxia inducible factor (HIF)-3α, BCL2/adenovirus E1B 19 kDa-interacting protein (BNIP)-3; sterol regulatory element-binding protein (SREBP)-1) genes were assessed in the lung, heart, liver, kidney, and spleen of rats administered vehicle and exposed to 0.8 ppm ozone or administered corticosterone (10 mg/kg body weight) and exposed to air (n = 5/group). A log2 transformation was used to linearize data. Results were regressed and compared using Pearson correlations. FC, fold-change relative to vehicle air group.

To independently verify glucocorticoid action in the regulation of genes modulated by ozone, air-exposed animals were administered exogenous corticosterone and expression profiles were compared with gene profiles of ozone-exposed animals. Genes implicated in inflammatory (GILZ, CCL2, IL-1β, TNF) and metabolic (HIF-3α, BNIP3, SREBP-1) responses were selected on the basis of exhibiting ozone-induced differential expression that was prevented by metyrapone in at least some organs. Overall, the pattern of response to exogenous corticosterone was similar to the response to ozone (Figure 5), with significant positive Pearson correlations in the lungs (*r* = 0.84, *P* = .02), heart (*r* = 0.94, *P* = .002), liver (*r* = 0.87, *P* = .01), and spleen (*r* = 0.88, *P* = .01), but not kidney (*r* = −0.26, *P* = .57).

## DISCUSSION

Activation of stress responses, including stimulation of the HPA axis and catecholamine release via sympathetic nervous system activation, initiate a spectrum of metabolic and immune responses. Although rapid and transient activation of the stress axis is a critical part of the adaptive response to stressors, chronic activation can precipitate adverse health outcomes, including metabolic, cardiovascular, neurobehavioral, and immune disorders (Anagnostis *et al.*, 2009; Chrousos and Kino, 2009). Effects of pollutants on the stress axis is therefore of interest in light of epidemiological observations of associations between air pollutants and these disease states. Consistent with our previous report (Thomson *et al.*, 2013), short-term exposure to ozone provoked a rapid increase in plasma corticosterone, which was eliminated in this study by pretreatment with metyrapone. The robust ozone-induced increase in plasma corticosterone coupled with elimination of this effect with metyrapone provides a model to assess the role of glucocorticoids in mediating biological effects of ozone inhalation. In contrast with effects in Wistar and Brown Norway rats (Bass *et al.*, 2013; Miller *et al.*, 2015), ozone inhalation did not cause a significant increase in circulating epinephrine levels in Fischer-344 rats. The reasons for this difference are unclear, but may relate to the strain and time course employed, as Fischer-344 rats have a hyper-responsive HPA axis (Dhabhar *et al.*, 1993) that could have resulted in different dynamics of sympathetic and humoral response to ozone.

The potential for ozone inhalation to cause lung injury and inflammation is well-established. In addition to direct effects of the gas, inflammatory mediators released by macrophages contribute to lung injury (Laskin *et al.*, 2010). An interstrain comparison showed that the lungs of Fischer rats are particularly resilient to ozone exposure, exhibiting reduced lung injury, neutrophil inflammation, and IL-6 compared with Sprague Dawley or Wistar rats (Dye *et al.*, 1999). In this study, Fischer rats administered metyrapone displayed an exaggerated pulmonary cytokine response to ozone compared to vehicle-exposed animals, suggesting that endogenous glucocorticoids limit the inflammatory response to ozone in these animals. At both the mRNA and protein level, cytokines that exhibited little response to ozone inhalation in vehicle-treated animals were increased by ozone in animals administered the low dose of metyrapone, consistent with direct inflammatory responses to ozone and indirect systemic anti-inflammatory glucocorticoid action. At the high dose of metyrapone, baseline levels of cytokines were significantly elevated in air-exposed animals, indicating pronounced dysregulation. These results are in line with previous studies showing that endogenous glucocorticoids limit the nature and magnitude of inflammatory signaling in the lungs (Incerpi *et al.*, 2015; Josephson *et al.*, 2012; Zhang *et al.*, 2009). The magnitude of inflammatory responses to ozone differs between individuals (Holz *et al.*, 1999). Factors such as genetic variability, age, and diet may explain some of the variance in inflammatory response and other health outcomes associated with ozone exposure (Vinikoor-Imler *et al.*, 2014). Our results suggest that intrinsic differences in stress axis function could also play a role in regulating the extent of lung inflammation in response to pollutant exposure.

Systemic inflammation, as indicated by increases in plasma cytokines, has been proposed as an important mechanism underlying cardiovascular and metabolic impacts of pollutant exposure, primarily on the basis of data from experimental exposure of animals to particulate matter (Rajagopalan and Brook, 2012). Evidence for effects of ozone on circulating cytokines has been mixed: controlled exposure of human volunteers to 0.3 ppm ozone for 2 h increased IL-8 (Devlin *et al.*, 2012), while little effect was observed in several other studies examining the cytokine response to ozone in humans and rodents (Bass *et al.*, 2013; Miller *et al.*, 2015; Urch *et al.*, 2010). We surveyed 24 plasma cytokines and found little evidence of an acute proinflammatory response to ozone in Fischer rats. However, there appeared to be a greater cytokine response to ozone in animals administered metyrapone. These results appear consistent with a role for endogenous glucocorticoids in regulating systemic inflammatory responses (Sternberg *et al.*, 1989), and suggest that responsiveness of the HPA axis could be relevant in determining the nature and extent of systemic inflammatory responses to pollutants.

Glucocorticoids exert well-characterized effects on metabolism, including permissive effects on glucagon and inhibition of insulin action that result in impaired peripheral glucose uptake and insulin resistance (Kuo *et al.*, 2015). In this work, ozone inhalation altered the levels of several hormones involved in regulating energy balance, notably reducing glucagon and ghrelin levels, and tended to decrease insulin. Administration of metyrapone modified acute effects of ozone on glucagon and insulin levels, but did not alter the effect of ozone on ghrelin. The pattern of response was complex, however, possibly reflecting the fact that these are single measurements of factors involved in a dynamic system, responsive to each other and to other stimuli in addition to corticosterone. The trend toward decreased insulin immediately after exposure is consistent with acute effects described after 6 h exposure of Brown-Norway and Wistar rats (Bass *et al.*, 2013; Miller *et al.*, 2015), but contrasts with the increase in plasma insulin measured in Wistar rats after a 16 h exposure (Vella *et al.*, 2015). The acute effect of ozone on ghrelin (decreased) is in line with an acute stress response that suppresses appetite; in the longer term, chronic stress and dysregulation of ghrelin and leptin can lead to stimulation of eating behavior and visceral fat accumulation (Sominsky and Spencer, 2014). PAI-1, an antifibrinolytic protein associated with exposure to ozone and particulate matter (Chuang *et al.*, 2007) and implicated in thrombosis, atherogenesis, and metabolic syndrome (Alessi and Juhan-Vague, 2006), was increased by ozone, consistent with stress-induced activation of factors implicated in hypercoagulability (Yamamoto *et al.*, 2002). Although little is known about the effect of chronic exposure to pollutants on hormones that regulate energy balance, long-term exposure of rodents to air pollutants has been shown to increase visceral fat accumulation, insulin resistance, and reward-seeking behavior (Allen *et al.*, 2013; Sun *et al.*, 2009). Exposure to high pollution levels in Mexico City was associated with decreased glucagon and ghrelin in children (Calderon-Garciduenas *et al.*, 2015), suggesting some commonality in the effects of chronic exposure of humans and those seen in the present short-term exposure of experimental animals.

Glucocorticoids, as the main effectors of the HPA axis, exert their effects primarily through receptor-mediated modulation of a number of biological pathways, notably regulating immune function and inflammation, carbohydrate and lipid metabolism, as well as the actions of other hormones (Chrousos and Kino, 2009). It has been reported by ourselves and others that inflammatory gene expression in extrapulmonary organs tends to be inhibited following ozone inhalation (Aibo *et al.*, 2010; Miller *et al.*, 2015; Thomson *et al.*, 2013). Here, we show that the reduction in transcript levels of inflammatory genes following ozone inhalation is blocked by metyrapone, demonstrating a key role for glucocorticoids in mediating these effects. Supporting this notion, GILZ, an important mediator of the immunosuppressive actions of glucocorticoids (Ayroldi and Riccardi, 2009), was induced by ozone in a glucocorticoid-dependent manner in most organs. Induction of inflammatory cytokine gene expression in renal epithelial cells has been shown to be insensitive to glucocorticoids (de Haij *et al.*, 2003), which may explain why cytokine gene expression tended to differ in the kidney compared with other organs. Metyrapone treatment also prevented effects of ozone on several genes implicated in glucose and lipid metabolism and homeostasis, consistent with a role for corticosterone in mediating a subset of tissue-level metabolic effects of pollutant exposure. Importantly, exogenous corticosterone reproduced effects of ozone exposure on inflammatory and metabolic genes that had been blocked by metyrapone. These results clearly demonstrate involvement of corticosterone in the regulation of inflammatory and metabolic genes in target tissues, consistent with the metyrapone results, and independent of potential nonspecific effects of metyrapone.

Tissue-level and systemic effects of metyrapone treatment did not always strictly correlate with plasma corticosterone levels. For example, the high dose of metyrapone was associated with considerably higher baseline cytokine mRNA and protein levels despite little difference in plasma corticosterone when compared with vehicle-exposed animals. In addition to inhibiting 11β-hydroxylase, metyrapone inhibits 11β-hydroxysteroid dehydrogenase type 1 (Sampath-Kumar *et al.*, 1997), which catalyses peripheral conversion of the inactive precursor 11-dehydrocorticosterone to corticosterone. As a result, impacts of metyrapone on tissue corticosterone and corticosterone-dependent transcriptional processes may be greater than predicted by plasma corticosterone. The time course of events should also be considered: changes in tissue mRNA levels will follow earlier changes in plasma and tissue corticosterone levels that may have been of greater magnitude due to rapid and transient effects of ozone and metyrapone on corticosterone. Furthermore, the complexity of the response across organs is consistent with involvement of other factors (eg target cell receptor density, enzymatic glucocorticoid metabolism) in defining organ-specific effects of ozone that are mediated by corticosterone, and with involvement of other signaling pathways. Effects of ozone on expression of the antioxidant and glucocorticoid-responsive MT genes were not prevented by metyrapone treatment in any organ, suggesting regulation by oxidative stress or other factors (Ghoshal and Jacob, 2001). Indeed, activation of proinflammatory genes in the kidney coupled with glucocorticoid-independent increases in MT genes is consistent with oxidant actions, which in turn may be implicated in metabolic effects of ozone inhalation (Vella *et al.*, 2015). Given the critical interrelationship among endocrine, inflammatory, antioxidant, and autonomic processes, it is probable that multiple interdependent mechanisms underlie systemic impacts of ozone exposure.

Exposure to a variety of different stressors, including ozone and particulate matter (Watkinson *et al.*, 2001), has been shown to elicit a stereotypical stress response in rodents that includes hypothermia and changes to cardiac electrophysiology. It is noteworthy that acute administration of corticosterone produces a phenotype that shares several features with the rodent response to inhaled pollutants, including reduced body temperature, altered blood glucose metabolism, and impacts on immune cells (Kainuma *et al.*, 2009). Our results demonstrate both glucocorticoid-dependent and -independent effects of ozone on metabolic and inflammatory processes in Fischer-344 rats, providing a model to investigate the role of glucocorticoids in mediating downstream effects of pollutant exposure. Stress-mediated metabolic and neurobehavioral dysfunction is well established, with glucocorticoids thought to play a central role (Chrousos and Kino, 2009). In addition to associations between pollutants, perceived stress (Mehta *et al.*, 2015), and stress-related diseases (eg cardiovascular disease, metabolic syndrome, depression), exposure to societal stressors such as violence and poverty is associated with increased risk of adverse health effects of air pollutants (Clougherty *et al.*, 2007), indicating that chronic stressors may contribute to vulnerability. The heightened pulmonary and systemic inflammatory signaling observed in response to ozone in metyrapone-treated animals implies that interindividual differences in HPA reactivity may be important in determining the nature and extent of inflammatory response to pollutant exposure, and thus contribute to susceptibility to inhaled pollutants. The degree to which these effects that follow acute exposure relate to the progression of disease states associated with chronic exposure to pollutants remains to be determined.

## Supplementary Material

Supplementary Data
