# Supplementary figures and images for: Ozone Inhalation Provokes Glucocorticoid-Dependent and -Independent Effects on Inflammatory and Metabolic Pathways
Source: Toxicol Sci. 2016 Apr 1;152(1):17–28. doi: 10.1093/toxsci/kfw061 (PMC12077420; doi:10.1093/toxsci/kfw061)

## Slide 1
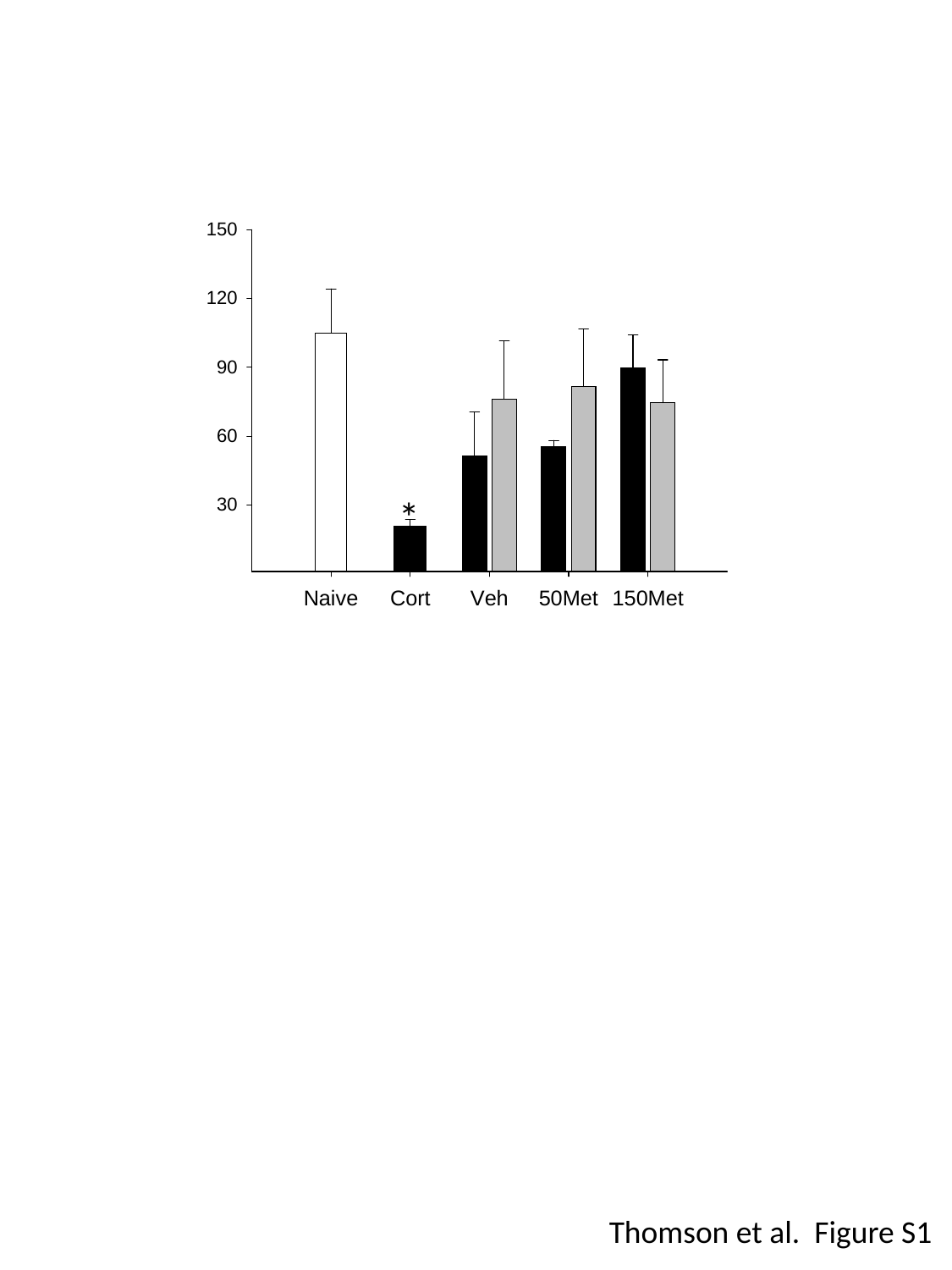

*
Thomson et al. Figure S1

Supplement: Supplementary Data [file toxsci_152_1_17_s1.zip › toxsci-16-0081-File007.pptx]

## Slide 1
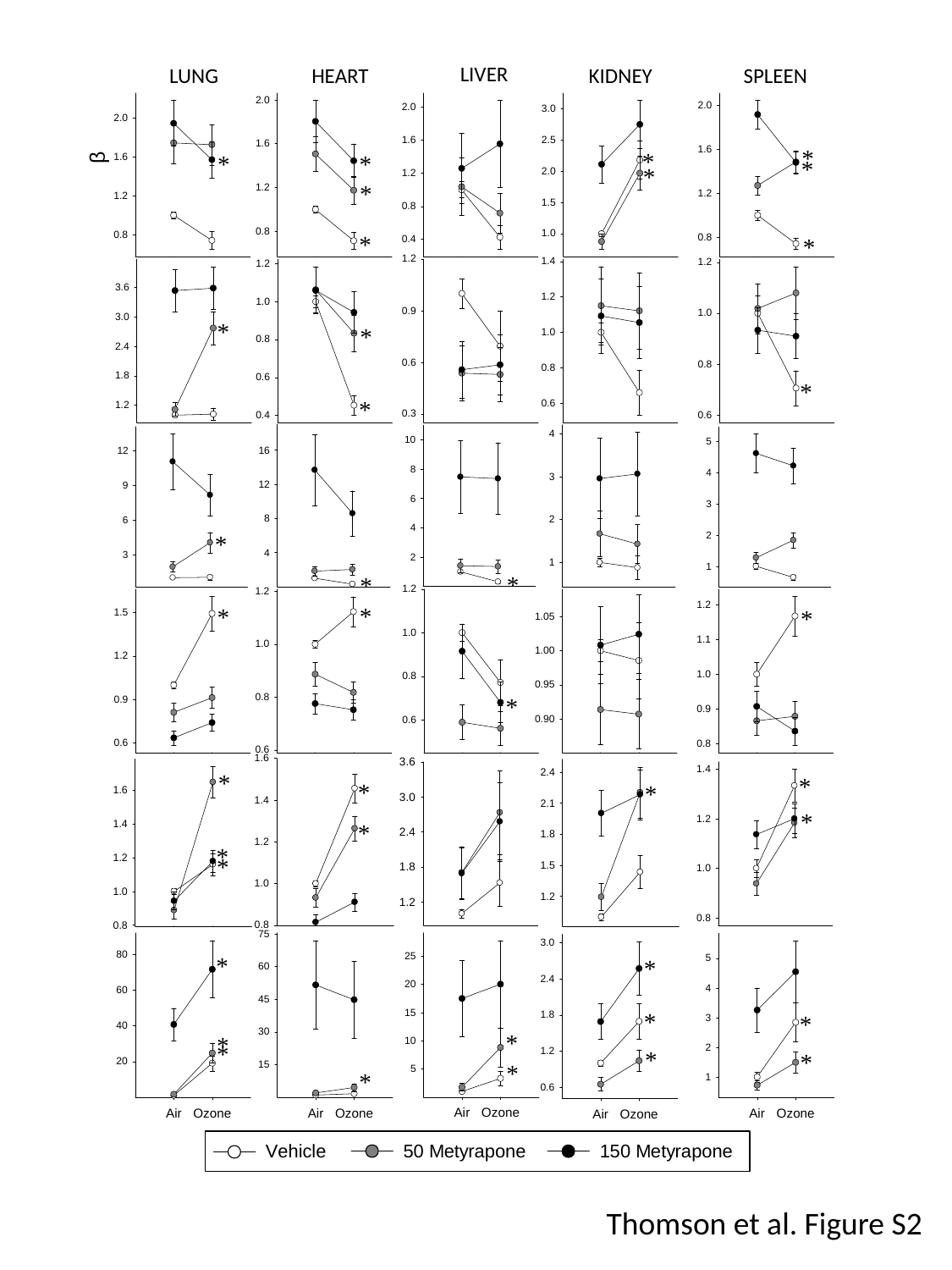

LIVER
LUNG
HEART
KIDNEY
SPLEEN
β
*
*
*
*
*
*
*
*
*
*
*
*
*
*
*
*
*
*
*
*
*
*
*
*
*
*
*
*
*
*
*
*
*
*
*
*
*
*
*
Thomson et al. Figure S2

Supplement: Supplementary Data [file toxsci_152_1_17_s1.zip › toxsci-16-0081-File008.pptx]
