# Supplementary material for: Ozone Inhalation Provokes Glucocorticoid-Dependent and -Independent Effects on Inflammatory and Metabolic Pathways
Source: Toxicol Sci. 2016 Apr 1;152(1):17–28. doi: 10.1093/toxsci/kfw061 (PMC12077420; doi:10.1093/toxsci/kfw061)
Supplement: Supplementary Data [file toxsci_152_1_17_s1.zip › toxsci-16-0081-File009.doc]

| **Primer**  **(Accession #)** | **Sequence** | **Amplicon size (bp)** |
| --- | --- | --- |
|  |  |  |
| **MT-1**  **(**NM_138826.4) | Sense (198-): atgtgcccagggctgtgt  Antisense (273-): gcagcactgttcgtcacttca | 76 |
| **MT-2**  (NM_001137564.1) | Sense (138-): tgcacctcctgcaagaaaa  Antisense (232-): cacttgtccgaagcctcttt | 95 |
| **HIF-3α**  (NM_022528.2) | Sense (1115-): ggtatcccaggcaacagtct  Antisense (1235-): gcaggtctgggctacaaaaa | 121 |
| **HMOX-1**  (NM_012580.2) | Sense (699-): gtcaagcacagggtgacaga  Antisense (770-): ctgcagctcctcaaacagc | 72 |
| **IL-1β**  (NM_031512.2) | Sense (133-): acagaccccaaaagattaagga  Antisense (225-): cgagatgctgctgtgagatt | 93 |
| **TNF**  (NM_012675.3) | Sense (347-): tccctctcatcagttccatgg  Antisense (448-): ctgcttggtggtttgctacg | 102 |
| **CCL2**  (NM_031530.1 ) | Sense (26-): cagaaaccagccaactctca  Antisense (169-): gtggggcattaactgcatct | 144 |
| **GILZ**  (NM_031345.1) | Sense (296-): gggatgtggtttccgttaaac  Antisense (395-): tgattcttcactagatccatggc | 100 |
| **SREBP-1 (**NM_001276707.1 **\|**  NM_001276708.1**)*** | Sense (1004-): acaagattgtggagctcaagg Antisense (1075-): tgcgcaagacagcagattta | 72 |
| **BNIP-3** (NM_053420.3) | Sense (577-): gtctcatctgttagccattgga  Antisense (664-): cagatccaagtctcatcaaaaggt | 88 |

* There are two mRNA transcript variants for SREBP-1. The primer set can bind to both variants.
